# Sterol-Sensing Domain (SSD)-Containing Proteins in Sterol Auxotrophic *Phytophthora capsici* Mediate Sterol Signaling and Play a Role in Asexual Reproduction and Pathogenicity

**DOI:** 10.1128/spectrum.03797-22

**Published:** 2023-01-11

**Authors:** Weizhen Wang, Zhaolin Xue, Linfang Xie, Xin Zhou, Fan Zhang, Sicong Zhang, Francine Govers, Xili Liu

**Affiliations:** a Department of Plant Pathology, College of Plant Protection, China Agricultural University, Beijing, People’s Republic of China; b Laboratory of Phytopathology, Wageningen University & Research, Wageningen, The Netherlands; c Institute of Crop Protection, College of Agriculture, Guizhou University, Guiyang, People’s Republic of China; Universita degli Studi del Molise

**Keywords:** *Phytophthora*, sterol-sensing domain, SSD-containing proteins, sterol signaling, asexual reproduction, pathogenicity

## Abstract

*Phytophthora* species are devastating filamentous plant pathogens that belong to oomycetes, a group of microorganisms similar to fungi in morphology but phylogenetically distinct. They are sterol auxotrophic, but nevertheless exploit exogenous sterols for growth and development. However, as for now the mechanisms underlying sterol utilization in *Phytophthora* are unknown. In this study, we identified four genes in *Phytophthora capsici* that encode proteins containing a sterol-sensing domain (SSD), a protein domain of around 180 amino acids comprising five transmembrane segments and known to feature in sterol signaling in animals. Using a modified CRISPR/Cas9 system, we successfully knocked out the four genes named *PcSCP1* to *PcSCP4* (for *P. capsici* SSD-containing protein 1 to 4), either individually or sequentially, thereby creating single, double, triple, and quadruple knockout transformants. Results showed that knocking out just one of the four *PcSCPs* was not sufficient to block sterol signaling. However, the quadruple “all-four” *PcSCPs* knockout transformants no longer responded to sterol treatment in asexual reproduction, in contrast to wild-type *P. capsici* that produced zoospores under sterol treatment. Apparently, the four PcSCPs play a key role in sterol signaling in *P. capsici* with functional redundancy. Transcriptome analysis indicated that the expression of a subset of genes is regulated by exogenous sterols via PcSCPs. Further investigations showed that sterols could stimulate zoospore differentiation via PcSCPs by controlling actin-mediated membrane trafficking. Moreover, the pathogenicity of the “all-four” PcSCPs knockout transformants was significantly decreased and many pathogenicity related genes were downregulated, implying that PcSCPs also contribute to plant-pathogen interaction.

**IMPORTANCE** *Phytophthora* is an important genus of oomycetes that comprises many destructive plant pathogens. Due to the incompleteness of the sterol synthesis pathway, *Phytophthora* spp. do not possess the ability to produce sterols. Therefore, these sterol auxotrophic oomycetes need to recruit sterols from the environment such as host plants to support growth and development, which seems crucial during pathogen-plant interactions. However, the mechanisms underlying sterol utilization by *Phytophthora* spp. remain largely unknown. Here, we show that a family of sterol-sensing domain-containing proteins (SCPs) consisting of four members in *P. capsici* plays a key role in sterol signaling with functional redundancy. Moreover, these SCPs play a role in different biological processes, including asexual reproduction and pathogenicity. Our study overall revealed the multiple functions of PcSCPs and addressed the question of how exogenous sterols regulate the development of heterothallic *Phytophthora* spp. via SSD-containing proteins.

## INTRODUCTION

Oomycetes, a class including many plant and animal pathogens, are filamentous microorganisms that morphologically resemble fungi and occupy similar ecological niches (1, 2). Filamentous plant pathogen is a collective term for fungi and oomycetes that infect plants. They often exploit similar mechanisms to invade and colonize their hosts, and to suppress host immunity by utilizing effectors (3). Despite these similarities, oomycetes and fungi are phylogenetically distinct; they evolved independently and belong to the supergroups Stramenopila and Opisthokonta, respectively (4, 5). In agricultural systems, disease control in crops remains a major challenge. Over the past few decades, fundamental research on effectors, effector targets, and resistance genes has opened new opportunities for more targeted and more efficient breeding of resistant plant varieties (6). Nevertheless, for many crops disease control still heavily relies on pesticide spraying, and therefore gaining deeper insight into fundamental biological processes in plant pathogens and recognizing the differences between oomycetes and fungi is of great significance for designing effective management strategies.

The differences between oomycetes and fungi have been noted from various perspectives (7). Notably, one of the differences is in the ability of each to synthesize sterols. Fungi can produce large amounts of ergosterol that serves as building block for membranes. Due to its importance for plasma membrane integrity, the interruption of ergosterol biosynthesis results in death of the fungus. CYP51, an enzyme that mediates a critical step in sterol biosynthesis, is the most important target of sterol synthesis inhibitors (SBIs), fungicides commonly used to combat fungal infections in humans and to control plant diseases (8). In oomycetes, however, sterol biosynthesis ability has evolved divergently among species (9). Only a few species in the Saprolegniales have been reported to synthesize sterols themselves, but all known species in the Perenosporales have lost this ability (9–11).

Within the Peronosporales *Phytophthora* is one of the best studied genera. It comprises over 190 described species many of which cause destructive diseases on a broad variety of plants and are easy to culture *in vitro* (12). Although *Phytophthora* spp. are sterol auxotrophic, they can recruit sterols from the environment to support growth and development (13). For example, exogenous sterols can promote mycelial growth, sporangium formation, and zoospore production in heterothallic *Phytophthora* spp. (14, 15), while homothallic *Phytophthora* spp. mainly exploit exogenous sterols for mycelial growth and oospore formation (16, 17). This implies that *Phytophthora* spp. must possess intracellular sterol signaling networks, likely involving intracellular transport and homeostasis of sterols, and signal transduction mediated by sterols. Interestingly, four sterol-sensing domain (SSD)-containing proteins (SCPs) have been identified in *Phytophthora capsici* and *Phytophthora sojae* based on genome data analyses (9, 18).

The SSD is around 180 amino acids in length and contains five transmembrane segments. It is present in multiple proteins that play key roles in sterol homeostasis and sterol signaling in humans, animals, and yeasts (19–21). Examples of SCPs are HMGCR (3-hydroxy-3-methylglutaryl coenzyme A-reductase), SCAP (the sterol regulatory element-binding protein [SREBP]-cleavage activating protein), NPC1 (Niemann-Pick disease type C1 protein), PTC (Patched protein), DISP (Dispatched protein), and PTR (PTC-related protein) (21, 22). It has been demonstrated that the SSD domain in human PTC is able to bind sterols, and that sterol-SSD binding or splitting can regulate downstream biological processes (23, 24). Moreover, the NPC1-like (NPC1L1) protein appears to be critical for intestinal cholesterol absorption and counter-balances hepatobiliary cholesterol excretion in animals (25, 26). As yet, the functions of SCPs in oomycetes have not been elucidated.

*Phytophthora capsici* is a typical heterothallic oomycete pathogen with a broad host range and significantly harms agricultural production (27). It reproduces via sexual and asexual life cycles. Because it is heterothallic, sexual reproduction requires two strains with different mating types to produce oospores, spores that can survive outside the plant under harsh conditions. Asexual reproduction is particularly relevant for the spread of this pathogen in the field. In the asexual life cycle, branched sporangiophores that emerge from hyphae produce sporangia, and the mature sporangia can quickly release motile zoospores that swim chemotactically to infect hosts (15). With the release of a genome sequence and the maturation of genetic manipulation techniques, *P. capsici* has gradually become a model species in oomycete research (28, 29). In this study, we used a modified CRISPR/Cas9 system to successfully knock out the four genes named *PcSCP1* to *PcSCP4* (for *P. capsici* SSD-containing protein 1 to 4) in *P. capsici*, either individually or sequentially, thereby creating single, double, triple, and quadruple knockout transformants. By investigating the biological characteristics of the knockout transformants, as well as their transcriptome responses to sterol and their internal actin cytoskeleton dynamics, the functions of PcSCPs in *P. capsici* were elucidated and the mechanisms on how exogenous sterols might regulate the development of *P. capsici* were discussed.

## RESULTS

### Characterization of PcSCPs and their phylogenic relationship with other SCPs.

**FIG 1:**
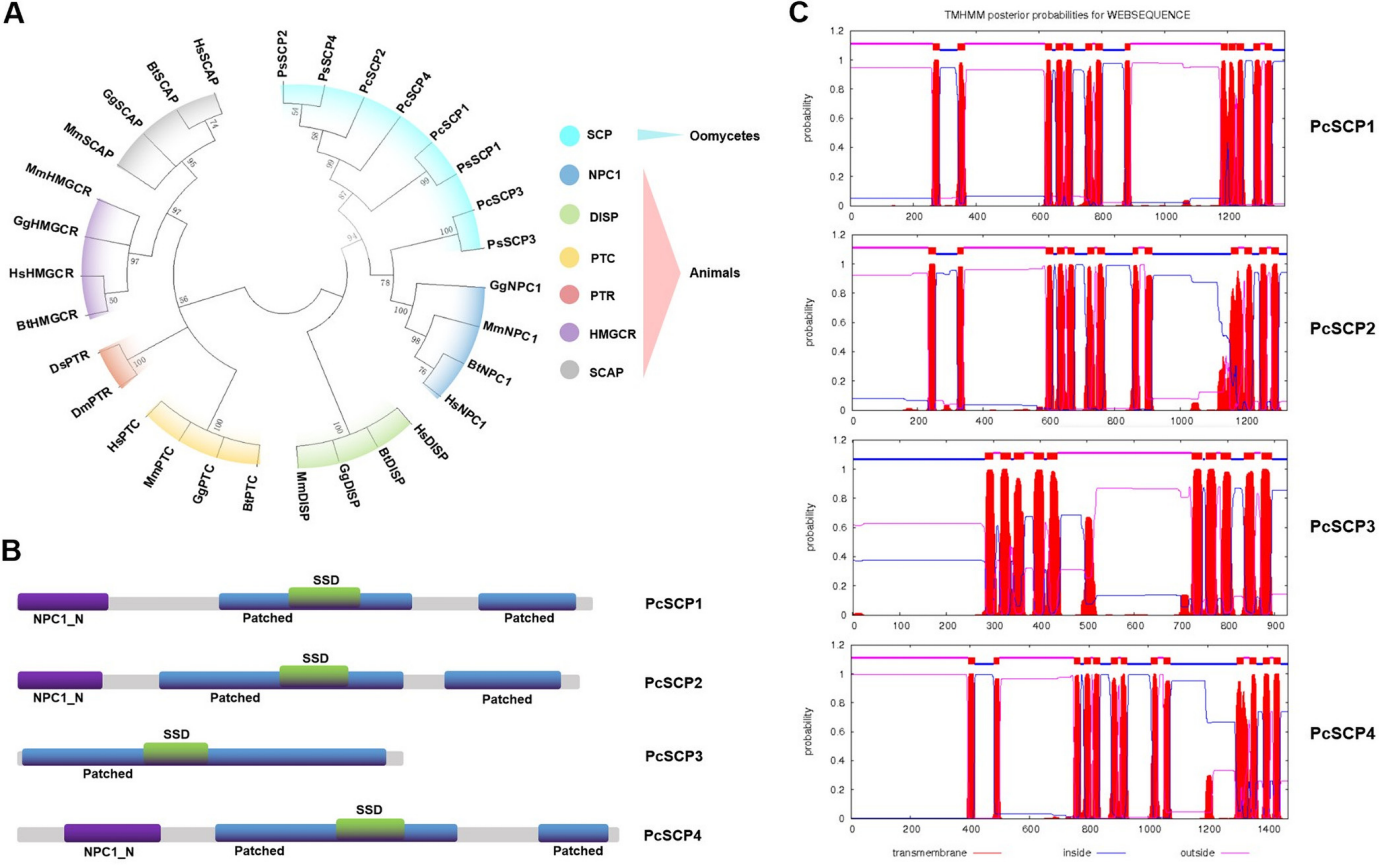
Characterization of PcSCPs and their phylogenic relationship with other SCPs from different eukaryotes. (A) Condensed molecular phylogenetic tree based on the amino acid sequences of SCPs from different organisms, including Homo sapiens (Hs), Bos taurus (Bt), Mus musculus (Mm), Gallus gallus (Gg), *P. capsici* (Pc), and *P. sojae* (Ps). Bootstrap values are expressed as percentages based on 1000 repetitions, and only those with ≥50% branch support are shown. (B) Functional domain organization of PcSCPs. Different domains are represented in different colors. (C) Transmembrane domain analysis of PcSCPs. Potential transmembrane domains are indicated by the red blocks on top.

Based on the analysis of the *P. capsici* genome, four genes encoding SCP proteins were identified, which were named PcSCP1 (protein ID in Joint Genome Institute [JGI] database: 543794), PcSCP2 (54680), PcSCP3 (563749), and PcSCP4 (506722). Using open reading frame (ORF) prediction and PCR (PCR) validation with the primers listed in Table S1, it was found that these four proteins are comprised of 1381, 1326, 927, and 1469 amino acids, respectively. Phylogenic analysis was conducted to characterize the relationship of PcSCPs with other SCPs from different protein families in animals and another *Phytophthora* species. The results showed that members of the same SCP family in different animal species cluster into a distinct phylogenic branch, indicating that SCPs are relatively conserved within the family (Fig. 1A). All *Phytophthora* SCPs are closely related to SCPs in the NPC1 family from animals (Fig. 1A), which are important players in sterol homeostasis and sterol signaling. Functional domain analysis showed that all these four PcSCPs contain SSD and Patched domains, and that PcSCP1, PcSCP2, and PcSCP4 also contain a NPC1_N domain (Fig. 1B). PcSCP3 lacks the NPC1_N domain that is a typical signature of NPC1 proteins (Fig. 1B), leading to a domain composition typical for Patched proteins. Therefore, it was predicted that PcSCP1, PcSCP2, and PcSCP4 are NPC1 related proteins, while PcSCP3 is a Patched-like protein. This is in agreement with the findings from a previous study analyzing the phylogeny of SCPs from *P. sojae* (18). Transmembrane domain prediction indicated that these four PcSCPs contain 13, 13, 10, and 13 transmembrane domains, respectively (Fig. 1C).

### The lack of any PcSCP gene does not block sterol signaling in *P. capsici*.

**FIG 2:**
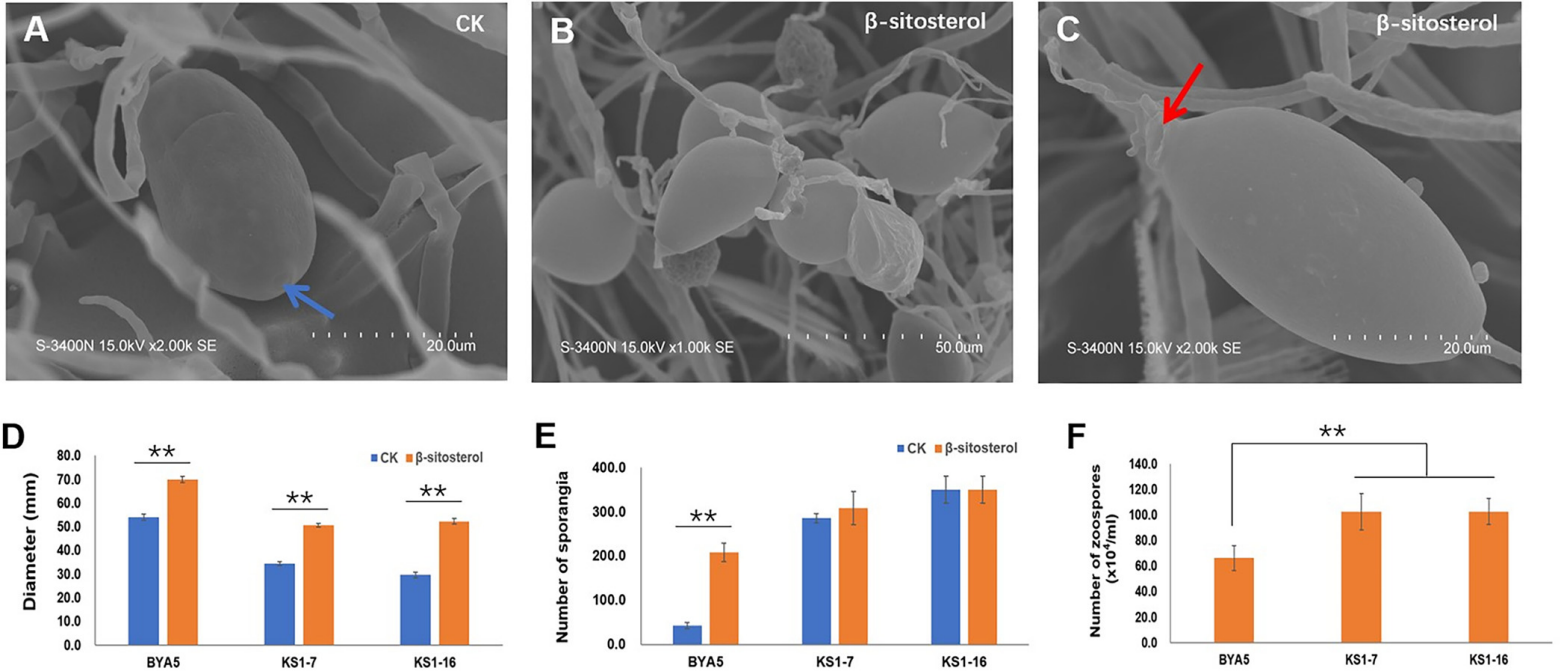
Effects of β-sitosterol on wild-type strain and *PcSCP1* knockout transformants of *P. capsici*. (A) The sporangium of the wild-type strain cultured on minimal medium without sterol (CK). The blue arrow indicates the closed zoospore release site. Clustered sporangia (B) and a single sporangium (C) of the wild-type strain cultured on minimal medium modified with 20 μg/mL β-sitosterol. The red arrow in (C) indicates the open zoospore release site. Mycelial growth (D) and sporangium production (E) of the wild-type strain and *PcSCP1* knockout transformants cultured on minimal medium without sterol (CK) or modified with 20 μg/mL β-sitosterol. (F) The zoospore production of the wild-type strain and *PcSCP1* knockout transformants cultured on the minimal medium modified 20 μg/mL β-sitosterol. Means with SD of three replicates are shown, and double asterisks denote a significant difference from each other (**, *P* < 0.01). BYA5 is the wild-type strain; KS1-7 and KS1-16 are *PcSCP1* knockout transformants.

Although *P. capsici* is a sterol auxotrophic oomycete, it can respond to sterol treatment. In particular, exogenous sterols promote mycelial growth, sporangium formation, and zoospore production in heterothallic *Phytophthora* spp. However, the mechanisms underlying these effects are largely unknown. To verify whether PcSCPs contribute to sterol signaling in *P. capsici*, we first knocked out the *PcSCP1* gene in *P. capsici*, using a modified CRISPR/Cas9 system (Table S2 and Fig. S1A). The wild-type strain (BYA5) and two homozygous gene knockout transformants (KS1-7 and KS1-16) were cultured on minimal medium with and without β-sitosterol, a prominent sterol component in plants and the V8 medium that is widely used for *Phytophthora* cultivation (30, 31). It showed that the wild-type strain BYA5 could grow without sterol but displayed a faster mycelial growth in the presence of sterol (Fig. 2A and D). Although *P. capsici* could produce a few sporangia on the minimal medium without sterol, these sporangia could not release zoospores with zoospore release site being closed (Fig. 2A). The addition of β-sitosterol significantly increased sporangium production and initiated zoospore release (Fig. 2B, C and E), resulting in zoospore production (Fig. 2F). *ΔPcSCP1* transformants could also respond to sterol treatment, as supplementary β-sitosterol promoted their mycelial growth and stimulated their zoospore production (Fig. 2D and F). However, it seems that PcSCP1 acts as a negative regulator of sporangium production, because *ΔPcSCP1* transformants produced more sporangia than BYA5 when cultured either with or without β-sitosterol (Fig. 2E). This was also reflected by a higher zoospore production in *ΔPcSCP1* transformants (Fig. 2F).

Similarly, the other three *PcSCPs* (*PcSCP2*, *PcSCP3* and *PcSCP4*) were also individually knocked out (Table S2 and Fig. S1A) and the resulting transformants’ responses to sterol treatment were determined. It was found that all single-gene knockout transformants grew faster in the presence of β-sitosterol; all strains also produced more sporangia and released zoospores under sterol treatment, although some transformants produced fewer zoospores under sterol treatment than the wild-type strain (Fig. S2). Taken together, these results indicate that the sterol signaling transduction pathways are not blocked in the single-gene knockout transformants.

### “All-four” *PcSCPs* knockout transformants are less sensitive to sterol treatment.

**FIG 3:**
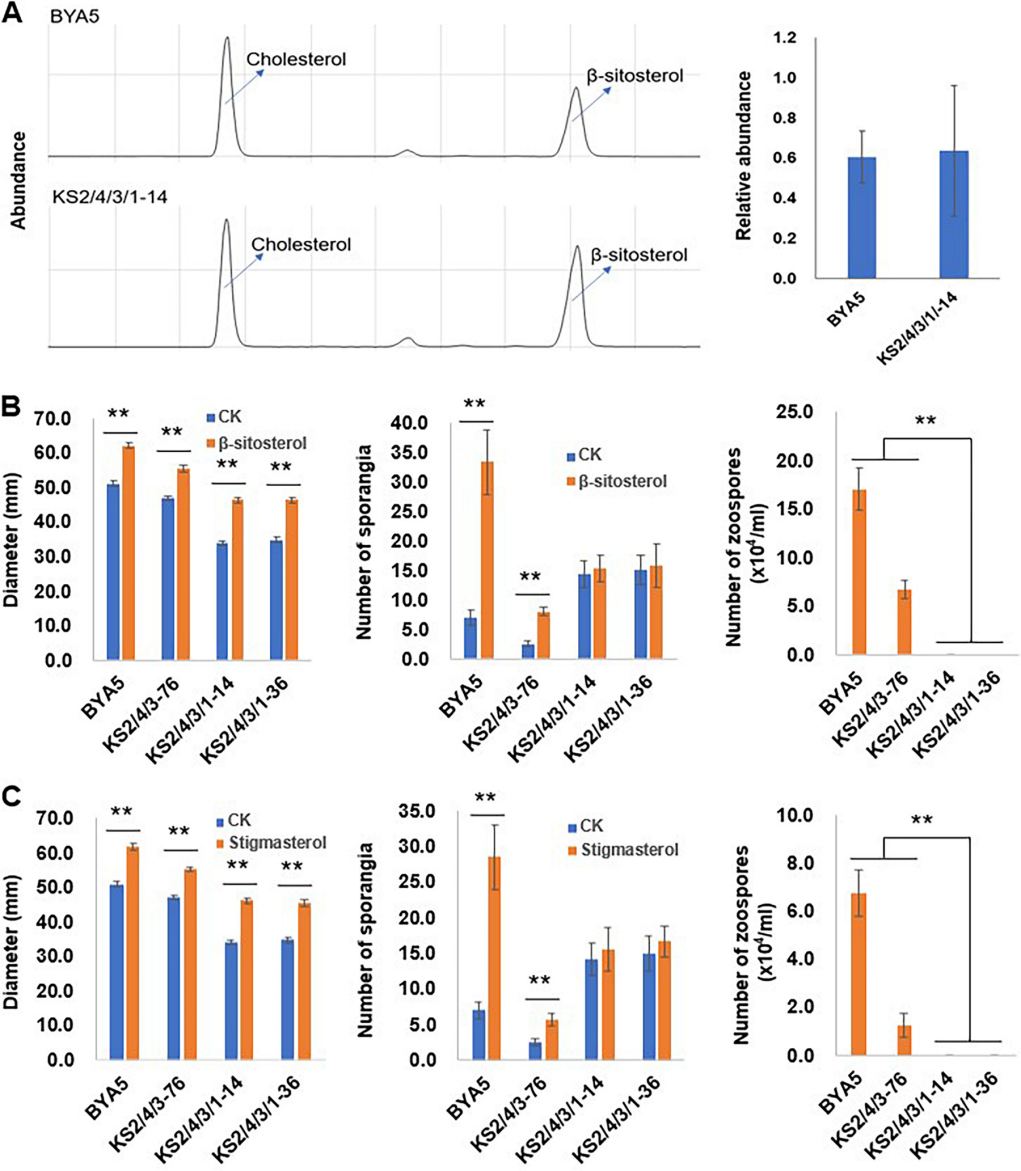
Sterol absorption and sterol response abilities of *P. capsici* wild-type strain and *PcSCPs* knockout transformants. (A) Sterol detection in mycelia of *P. capsici* wild-type strain and one *PcSCP2/4/3/1* knockout transformant after β-sitosterol treatment. The chromatograms of sterol detection from different strains are shown on the left. The relative amount of sterol absorbed is shown on the right. Cholesterol was used as the internal standard for β-sitosterol normalization. All of the sterols indicated in the figure were detected as sterol derivatives with a trimethylsilyl at the C-3 hydroxyl. Effects of β-sitosterol (B) and stigmasterol (C) on wild-type strain and *PcSCPs* knockout transformants of *P. capsici*. Means with SD of three replicates are shown, and double asterisks denote a significant difference (**, *P < *0.01). BYA5 is the wild-type strain; KS2/4/3-76 is a *PcSCP2/4/3* knockout transformant; KS2/4/3/1-14 and KS2/4/3/1-36 are the *PcSCP2/4/3/1* knockout transformants. CK means the strains were cultured without sterol treatment and zoospore production was measured under sterol (β-sitosterol or stigmasterol) treatment.

The phylogenic analysis showed that PcSCP2 and PcSCP4 are highly similar (Fig. 1), which suggests a potential redundancy in function between each other. Therefore, the *PcSCP4* gene was knocked out in the *PcSCP2* knockout transformant, thus obtaining double-gene knockout transformants (Table S2 and Fig. S1B). However, these *ΔPcSCP2/4* transformants could still respond to sterol treatment in vegetative growth and asexual reproduction, as their mycelial growth become faster, and they all were able to produce zoospores under sterol treatment (Fig. S3). The triple-gene knockout transformant was obtained by further modifying the *ΔPcSCP2/4* transformant, knocking out the *PcSCP3* gene (Table S2 and Fig. S1C). Likewise, the *PcSCP1* gene was knocked out in the triple-gene knockout transformant to obtain the “all-four” *PcSCPs* knockout transformants (Table S2 and Fig. S1D). To determine whether PcSCPs are important for the sterol uptake ability of *P. capsici*, the wild-type strain and one *ΔPcSCP2/4/3/1* transformant were treated with the same amount of β-sitosterol and then the sterol content within the mycelia was measured. Comparison revealed that the “all-four” *PcSCPs* transformant had a similar level of recruited sterol as the wild-type strain (Fig. 3A), indicating that PcSCPs are not important for sterol absorption of *P. capsici*. Sterol response evaluation showed that the *ΔPcSCP2/4/3* transformant could still respond to β-sitosterol and stigmasterol in vegetative growth and asexual reproduction, but the “all-four” *PcSCPs* knockout transformants (*ΔPcSCP2/4/3/1*) were less sensitive to sterol treatments (Fig. 3B and C). Although the *ΔPcSCP2/4/3/1* transformants grew faster under sterol treatments, they seemed incapable of responding to sterol treatments in asexual reproduction, as their sporangium formation did not increase and they could not produce zoospores under sterol treatments (Fig. 3B and C). Therefore, the PcSCPs show redundancy in functions but together they are important players in sterol signaling pathways that orchestrate asexual production, and these PcSCPs may serve as either sterol transporters or sensors. Because exogenous sterols could still promote the mycelial growth of *ΔPcSCP2/4/3/1* transformants, it is conceivable that sterols can regulate vegetative growth and asexual reproduction via different pathways.

### Sterols can regulate the expression of many genes via PcSCPs in *P. capsici*.

**FIG 4:**
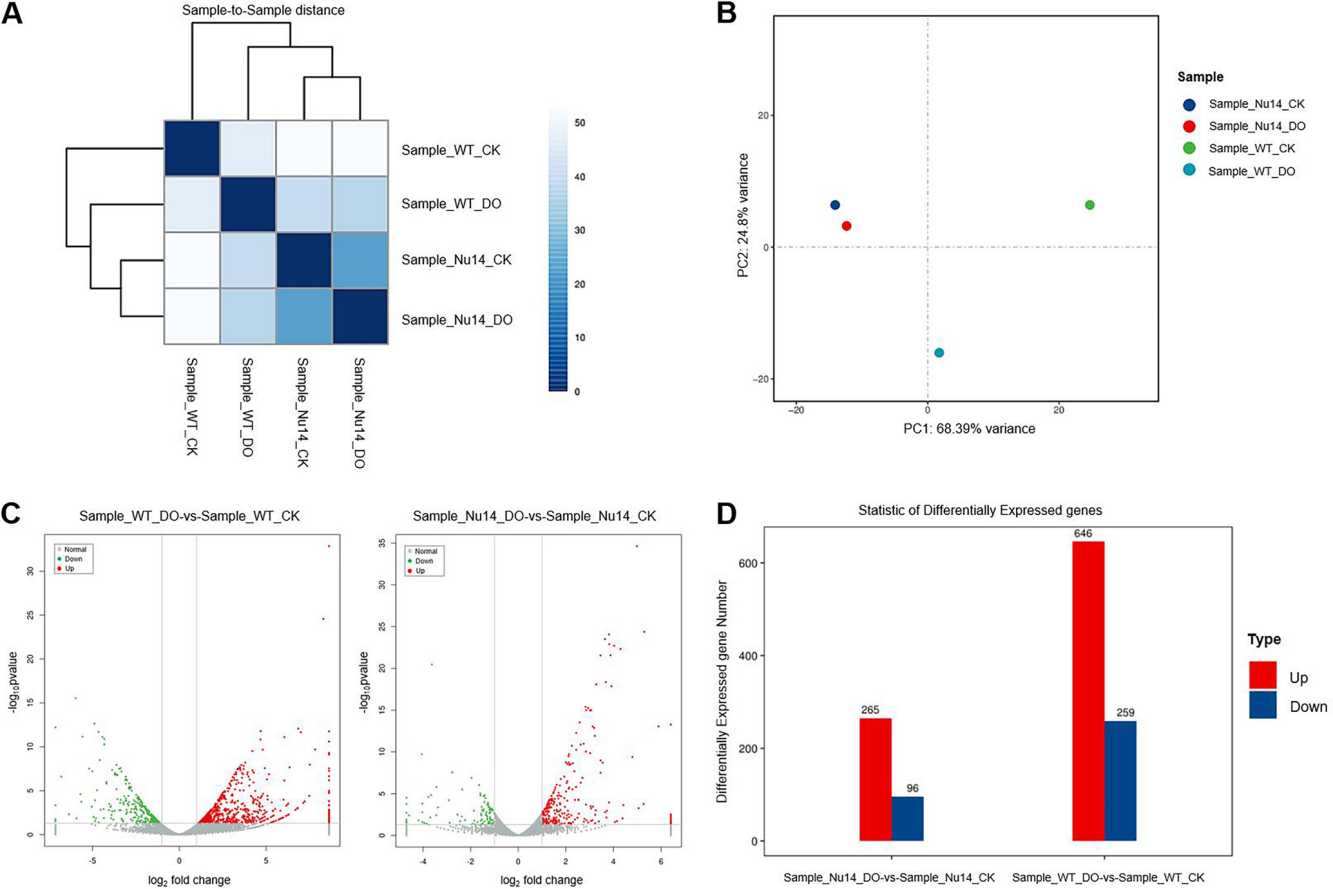
The effect of sterol treatment on transcriptomes of *P. capsici* wild-type strain and one *PcSCP2/4/3/1* knockout transformant. (A) Sample to sample distances of different strains with and without β-sitosterol treatment. (B) Principal-component analysis based on the transcriptomes of different strains with and without β-sitosterol treatment. (C) Volcano plot showing the fold of change (X-axis) of upregulated (red) and downregulated (green) genes and the statistical significance (Y-axis). (D) The number of genes that showed at least a 2-fold increase (up) or decrease (down) in expression in the two comparison groups. Sample_WT_CK indicates the wild-type strain BYA5 without sterol treatment; sample_WT_DO indicates the wild-type strain BYA5 under the treatment of 20 μg/mL β-sitosterol; sample_Nu14_CK indicates the *PcSCP2/4/3/1* knockout transformant KS2/4/3/1-14 without sterol treatment; sample_Nu14_DO indicates the *PcSCP2/4/3/1* knockout transformant KS2/4/3/1-14 under the treatment of 20 μg/mL β-sitosterol.

To investigate how exogenous sterols regulate the development of *P. capsici* and what pathways PcSCPs participate in during the regulation, RNA-seq was conducted for comparison of the transcriptomes of BYA5, the wild-type strain, with and without β-sitosterol treatment (Comparison Group 1: Sample_WT_DO-vs-Sample_WT_CK) and KS2/4/3/1-14, one of the “all-four” *PcSCPs* knockout transformants, with and without β-sitosterol treatment (Comparison Group 2: Sample_Nu14_DO-vs-Sample_Nu14_CK). It was found that the transcriptome of the wild-type strain changed significantly under sterol treatment, with 905 differentially expressed genes (DEGs), 646 of which being upregulated and 259 downregulated (Fig. 4). In contrast, the transcriptome of the *ΔPcSCP2/4/3/1* transformant showed far less changes under the same treatment, with only 361 DEGs, 265 of which being upregulated and 96 downregulated (Fig. 4). The transcriptome data were validated using qPCR analysis of 23 randomly selected genes that showed changes, including 18 genes in Comparison group 1 and five genes in Comparison Group 2. Most of the genes showed consistent expression patterns in the transcriptome and the qPCR analysis (Table S3), indicating that the transcriptome data were reliable. The transcriptome comparison further confirmed that the *ΔPcSCP2/4/3/1* transformants are less sensitive to sterol treatment. These results suggest that the expression of hundreds of genes is affected by exogenous sterols and point to involvement of PcSCPs in pathways regulating gene expression.

Based on GO enrichment analysis of the DEGs in the two comparison groups, it was found that upon sterol treatment most of the genes differently expressed in the wild-type strain (Comparison Group 1) are predicted to participate in various biological processes, including pathogenesis, defense response, carbohydrate metabolic process, cell communication, transmembrane transport, sphingolipid metabolic process, and lysosome organization. From a cellular component perspective, the DEGs encode proteins mainly localized in the extracellular region, membrane, clathrin adaptor complex, dynein complex, lysosome, integral component of membrane, membrane coat, endoplasmic reticulum, and microtubule associated complex (Fig. S4). Notably, the enrichment of several GO categories was less prominent in the *ΔPcSCP2/4/3/1* transformant (Comparison Group 2), especially for DEGs participating in pathogenesis and defense response as well as those localized in the extracellular region, clathrin adaptor complex, dynein complex, lysosome, and microtubule associated complex (Fig. S4).

### Exogenous sterols promote zoosporogenesis in *P. capsici* by remodeling the actin cytoskeleton via PcSCPs.

**FIG 5:**
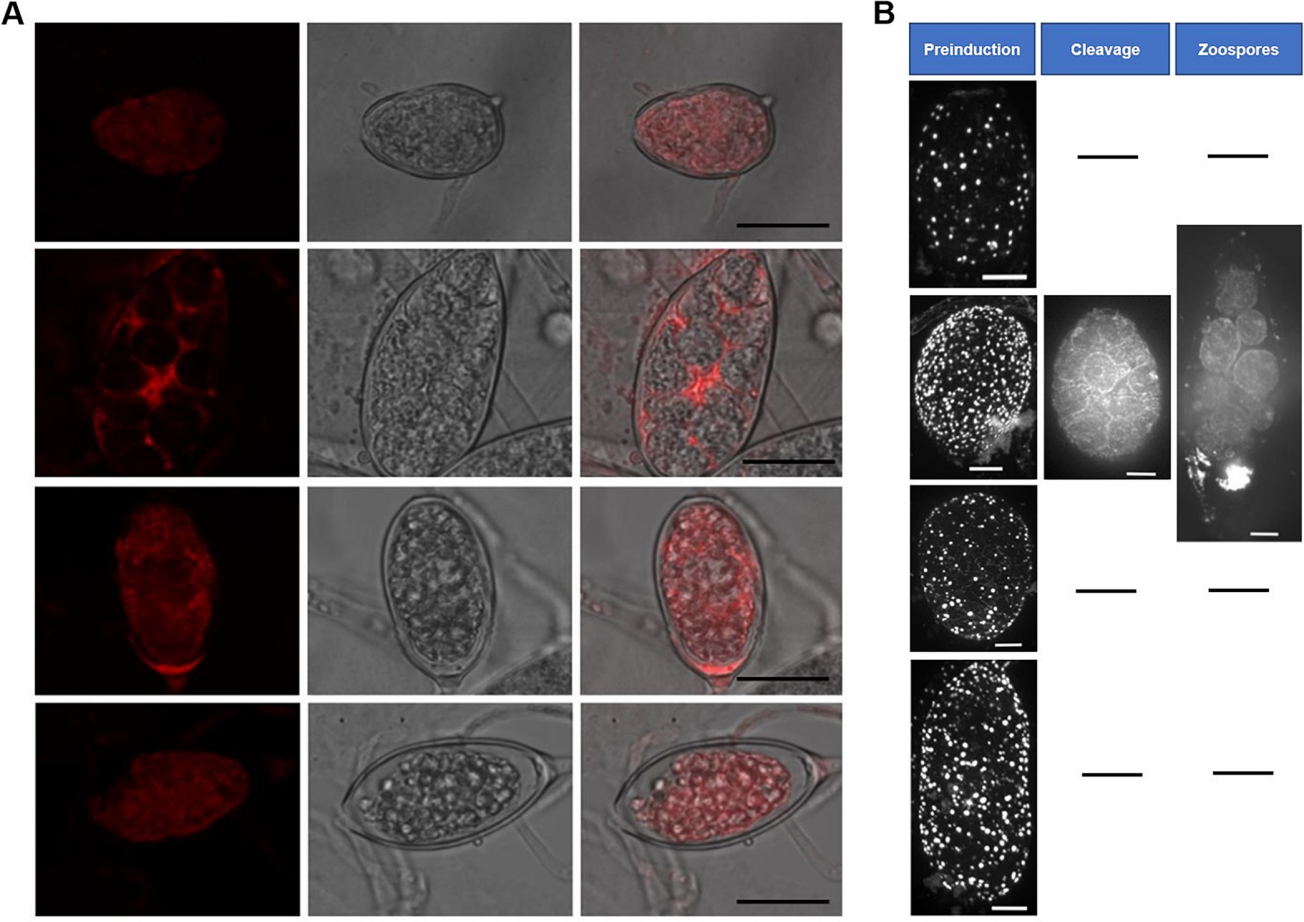
Membrane polarization and F-actin organization in sporangia of *P. capsici* wild-type strain and *PcSCP2/4/3/1* knockout transformants with and without sterol treatment. (A) FM4-64 staining of sporangia of the wild-type strain BYA5 (rows 1 and 2) and *PcSCP2/4/3/1* knockout transformant KS2/4/3/1-14 (rows 3 and 4) cultured without (rows 1 and 3) and with 20 μg/mL β-sitosterol (rows 2 and 4). Fluorescence (left), white (middle), and merged (right) fields are shown. Bar = 50 μm. (B) F-actin labeling with rhodamine-phalloidin in sporangia of the wild-type strain BYA5 (rows 1 and 2) and *PcSCP2/4/3/1* knockout transformant KS2/4/3/1-36 (rows 3 and 4) cultured without (rows 1 and 3) and with 20 μg/mL β-sitosterol (rows 2 and 4). The black horizontal lines indicate that cleavage and zoosporogenesis do not occur in the absence of sterol and in the *PcSCP2/4/3/1* knockout transformant. Bar = 10 μm.

Since exogenous sterols no longer promoted zoospore production of the *ΔPcSCP2/4/3/1* transformants, the possible mechanisms underlying this were investigated. The sporangia of the *P. capsici* wild-type strain and the *ΔPcSCP2/4/3/1* transformants formed under both conditions (with and without exogenous sterol) were stained with FM4-64. In the absence of sterol in the medium membranes were evenly distributed all over the sporangia of both the wild-type strain and the “all-four” knockout transformants. When cultured in the medium with β-sitosterol membrane polarization was observed in the sporangia of the wild-type strain, but not in the sporangia of the *ΔPcSCP2/4/3/1* transformant (Fig. 5A). This suggests that exogenous sterols can promote zoosporogenesis by regulating membrane dynamics in sporangia and that PcSCPs play a role in this process.

Actin cytoskeleton remodeling can affect a variety of cellular processes by providing the forces required for membrane dynamics (32, 33). It has also been demonstrated that actin plays an important role in sporangium development in *Phytophthora cinnamomi* (34). Moreover, latrunculin B, an inhibitor of actin polymerization, could significantly inhibit mycelial growth and zoospore production of *P. capsici* (Fig. S5). To test the hypothesis that exogenous sterols can remodel the actin cytoskeleton, we visualized the filamentous actin (F-actin) configuration in the sporangia of *P. capsici* with phalloidin staining (35). The results showed that F-actin was evenly distributed in the sporangia of wild-type *P. capsici* cultured without sterol while in the presence of β-sitosterol the F-actin aggregated orderly at the zoospore cleavage site during zoosporogenesis (Fig. 5B). Similar sterol-triggered F-actin aggregation patterns were observed in sporangia of another *Phytophthora* species, *Phytophthora palmivora*, in which F-actin was labeled with Lifeact-eGFP (36) (Fig. S6) and it is conceivable that the developmental process leading to zoosporogenesis is somewhat conserved within the *Phytophthora* genus. These findings suggest that F-actin is involved in membrane polarization and that exogenous sterols can facilitate sporangial development by regulating actin dynamics. However, in sporangia of the *ΔPcSCP2/4/3/1* transformant no F-actin aggregation was observed under any circumstance, with or without sterol treatment (Fig. 5B). Apparently, in the “all four” knockout transformant the actin dynamics was not influenced by exogenous sterols, suggesting that PcSCPs play an important role in sterol-regulated actin dynamics.

### “All-four” *PcSCPs* knockout transformants display reduced pathogenicity.

**FIG 6:**
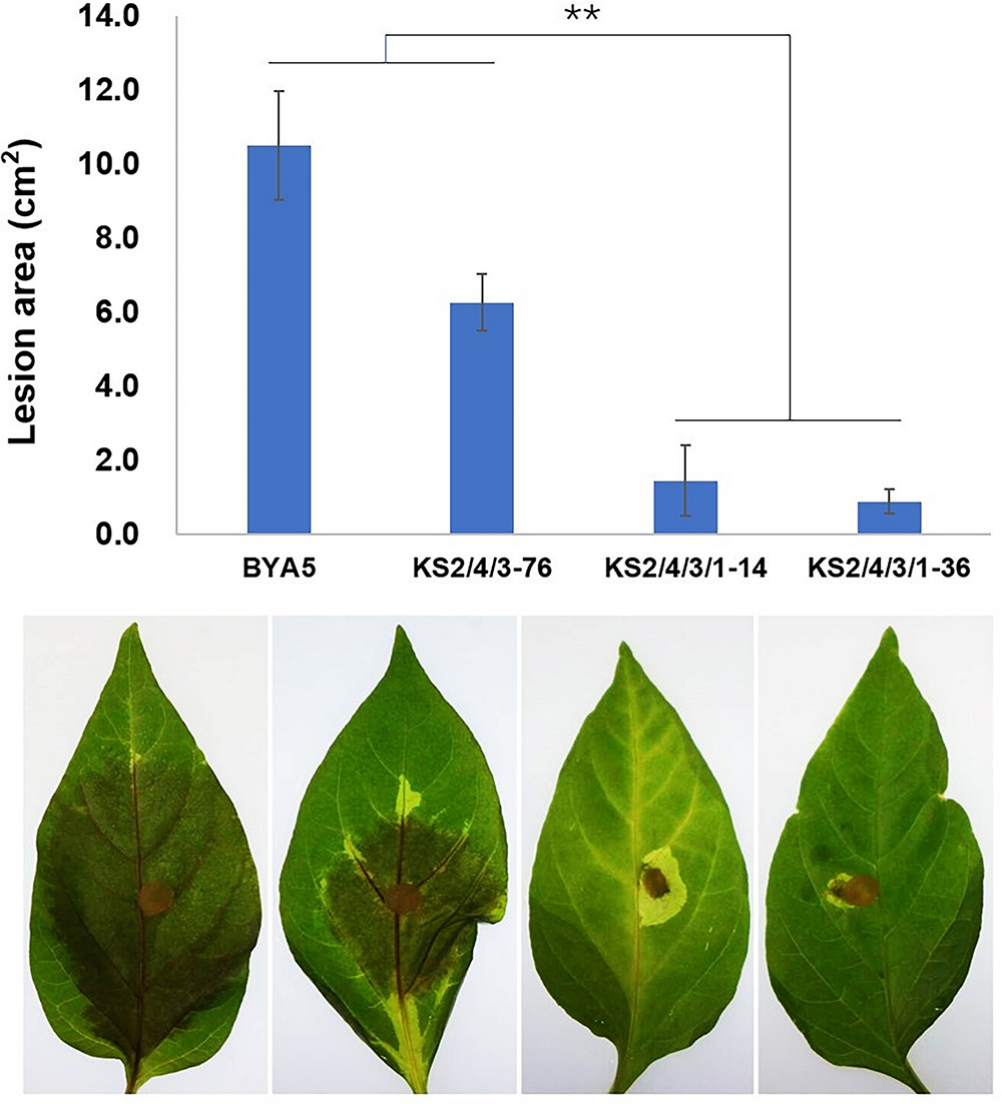
Pathogenicity evaluation of the wild-type strain and *PcSCPs* knockout transformants with pepper leaves. Detached pepper leaves were inoculated with mycelial plugs and the sizes of lesions as shown in upper panels were determined at 3 dpi. Means with SD of five replicates are shown, and double asterisks denote a significant difference (**, *P < *0.01). BYA5 is the wild-type strain; KS2/4/3-76 is the *PcSCP2/4/3* knockout transformant; KS2/4/3/1-14 and KS2/4/3/1-36 are the “all-four” *PcSCPs* knockout transformants.

The finding that the expression of many genes related to pathogenesis in the wild-type *P. capsici* changed under sterol treatment, but not in the *ΔPcSCP2/4/3/1* transformant, raised questions about the role of PcSCPs in pathogenicity. The pathogenicity was evaluated by inoculating detached pepper leaves with mycelial plugs of the various knockout transformants and the wild-type strain. The results showed that the pathogenicity of the single- and double-gene knockout transformants (*ΔPcSCP1*, *ΔPcSCP2*, *ΔPcSCP3*, *ΔPcSCP4*, and *ΔPcSCP2/4*) was comparable to that of the wild-type strain (Fig. S7). The triple-gene knockout transformant (*ΔPcSCP2/4/3*) was less pathogenic than the wild-type strain, but it could still cause obvious lesions on pepper leaves (Fig. 6). However, the “all-four” *PcSCPs* knockout transformants displayed dramatically reduced pathogenicity compared to the wild-type strain and the *ΔPcSCP2/4/3* transformant (Fig. 6). Further analysis of the transcriptome data were conducted by comparing the expression of genes with GO terms related to pathogenesis in the *ΔPcSCP2/4/3/1* transformant and those in the wild-type strain under the same conditions (with or without sterol). The analysis showed that 11 out of the 14 identified DEGs related to pathogenesis were significantly downregulated in the *ΔPcSCP2/4/3/1* transformant without sterol treatment and that all of the nine identified DEGs related to pathogenesis were significantly downregulated in the *ΔPcSCP2/4/3/1* transformant under the condition with sterol treatment (Table S4). This indicates that PcSCPs contribute to the pathogen-plant interaction process by regulating the expression of a series of pathogenesis related genes, which may, to some extent explain why the pathogenicity of the *ΔPcSCP2/4/3/1* transformant is decreased dramatically compared to that of the wild-type strain.

## DISCUSSION

Although most pathogenic oomycetes are sterol auxotrophic, it has been demonstrated that *Phytophthora* spp. can recruit exogenous sterols from the environment and use them for growth and development (9, 13). By supplementing the minimal medium with exogenous sterols, it was found that sterols can promote vegetative growth of *P. capsici* and stimulate asexual reproduction in this species (15). However, the mechanisms underlying these effects remain unknown. The SSD is a signature of many proteins that are important for sterol signal transduction pathways involved in sterol transport and sterol sensing in animals and yeasts (19, 23, 24). Interestingly, four genes encoding SSD-containing proteins (SCPs) were identified in different *Phytophthora* species (9, 18). In addition to the SSD these SCPs also contain the Patched domain and three of them contain the NPC1_N domain. Many SCPs in other organisms have similar domain organizations (37), suggesting that SCPs are relatively conserved in different lineages. As such, PcSCPs like other SCPs, may play a role in sterol related networks.

Using a modified CRISPR system, we successfully knocked out the *PcSCP* genes individually and sequentially and obtained single, double, triple, and quadruple knockout transformants. The lack of any *PcSCP* gene was not sufficient to block sterol signal transduction. However, the “all-four” *PcSCPs* knockout transformants could not respond to sterol treatment in asexual reproduction. Hence, some of these PcSCPs are functionally redundant but as a collective they are important players in sterol transduction pathways. Advances in plant genetics have benefited our understanding of the scenarios of synergistic phenotypes conferred by homologous genes. Some homologs may fail to complement each other or become dose-dependent in certain multiple-mutant combinations, suggesting that the degree of redundancy can vary (38). Based on phylogenetic analysis of SCPs, it could be noted that PcSCP2 and PcSCP4 are very closely related (Fig. 1A), and it is possible that these two proteins have a high function redundancy with each other. PcSCP3 is distinct from the others; the protein is shorter and lacks the NPC1_N domain. Knocking out of *PcSCP3* individually or on the basis of double-gene knockout strain had no obvious impact on biological characteristics, indicating that this gene may not be essential for *P. capsici*. This is consistent with the results of another study, where the ortholog of *PcSCP3* was individually knocked out in *P. sojae* (18). We also found some of the single-gene knockout transformants, when treated with sterol, produced fewer zoospores compared to the wild-type strain. It might be that the single protein has a partial contribution to sterol utilization for *P. capsici* or that the lack of one *PcSCP* leads to expression regulations of other *PcSCP* genes. Although sterol could promote vegetative growth and reproduction in the *PcSCP2/4/3* knockout transformants, the “all-four” *PcSCPs* knockout transformants totally lost the ability to produce zoospores under sterol treatment, indicating that PcSCP1 is redundant with some of the others and plays a key role in *P. capsici*. On the other hand, the “all-four” *PcSCPs* knockout transformants could still respond to sterol treatment in vegetative growth, suggesting that exogenous sterols promote mycelial growth and asexual reproduction of *P. capsici* by different mechanisms.

Because SCPs possess multiple transmembrane helices and some of them have a higher mobility, untangling their structures seems challenging. Although biochemical evidences have long before demonstrated that the SSD domain can directly bind to sterols (24), the working mechanisms of SCPs remained elusive until 2016 when structure determinations were performed supported by single-particle cryo-electron microscopy (39). Based on the molecular basis of the interaction between SCPs and sterols, it was suggested that SCPs be divided into two classes: moderator proteins and transporter proteins (37). Our results indicate that PcSCPs are important for *P. capsici* to utilize exogenous sterols, but it is still not clear whether they act as sterol moderators or sterol transporters in this pathogen.

By investigating the sporangial development process in *P. capsici*, we found that the maturation of sporangia in the wild-type strain can be induced by supplementing exogenous sterols. Vesicle trafficking and membrane polarization in sporangia are preconditions of zoospore differentiation, and sterols can significantly accelerate these processes. Transcriptome analysis showed that the expression of many genes encoding cytoskeleton-related proteins changed significantly upon sterol treatment, including components of clathrin adaptor complex, dynein complex, and microtubule associated complex. In eukaryotes, these proteins are known to have connection to one of the three cytoskeletal elements—actin (40–42). Moreover, it has been shown that the actin cytoskeleton is important for membrane trafficking in different eukaryotes and plays a role in the sporangial development in *P. cinnamomi* (32–34). Further investigations indicated that the dynamics of F-actin in the sporangia of wild-type *P. capsici* is indeed affected by exogenous sterols. However, the well-organized membrane polarization and F-actin reconfiguration were not observed in the “all-four” *PcSCPs* knockout transformants under sterol treatment. Therefore, exogenous sterols can possibly activate certain pathways via PcSCPs, thereby affecting the activity or functioning of actin related proteins, which subsequently control F-actin dynamics that influence membrane trafficking and zoospore differentiation. In addition, other asexual reproduction components that changed under sterol treatment may also contribute to these processes, including extracellular proteins and membrane proteins. A previous study found that dynein light chain 1 is essential for flagellate formation in *Phytophthora nicotianae* (43). Therefore, exogeneous sterols may also have a role in flagellate development of zoospores in *P. capsici*. Notably, the “all-four” *PcSCPs* knockout transformants displayed dramatically decreased pathogenicity compared with the wild-type strain, possibly due to lower expression of pathogenesis related genes in the transformants. This is in contrast to a previous finding that the SCP deficient *Toxoplasma* mutants are slightly more virulent in mice than the parental strain (44). Nevertheless, the mechanisms underlying pathogenicity regulation need to be further clarified. Given that in the absence of sterol in the medium the transcriptome of the “all-four” *PcSCPs* knockout transformant was notably divergent from that of the wild-type strain, it is likely that PcSCPs also function in some sterol independent pathways. Overall, this study revealed that PcSCPs are important for sterol signaling and have multiple functions involved in different biological processes, including asexual reproduction and pathogenicity.

## MATERIALS AND METHODS

### Growth conditions of *P. capsici* strains.

The *P. capsici* wild-type strain BYA5 was isolated from an infected pepper sample collected in Gansu province of China in 2011. The wild-type strain and transformants were routinely cultured on solid V8 medium at 25°C in the dark. For DNA and RNA isolation, the strains were cultured on solid V8 medium covered with one layer of cellophane (0.02 mm in thickness) at 25°C in the dark for several days, after which the mycelia were collected and stored at −80°C until required. For sterol treatment experiments, *P. capsici* strains were cultured on the minimal medium (0.1 g KNO_3_, 0.2 g K_2_HPO_4_, 0.1 g MgSO_4_, 0.1 g CaCl_2_, 0.1 g l-asparagine, 0.05 g l-serine, 4 g glucose and 1 mL trace elements [200 mg FeEDTA, 10 mg CuSO_4_, 10 mg MnCl_2_, 10 mg Na_2_MoO_4_, 10 mg Na_2_B_4_O_7_, 20 mg ZnSO_4_, and 100 mg thiamine hydrochloride in 100 mL of distilled water] in 1 L of distilled water) without sterols (45) for at least two rounds to completely deplete sterols in the mycelia. The strains were then transferred to the minimal medium modified with sterol (β-sitosterol or stigmasterol) solubilized in methanol (1% of the medium) to a final concentration of 20 μg/mL with three replicates, or to the minimal medium modified with only 1% methanol as a control. After 4 days of cultivation, the colony diameters were measured. For asexual reproduction ability evaluation, the strains were cultured at 25°C in the dark for 4 days and then in a 12 h-light/12 h-dark photoperiod for another 6 days. The sporangium production was measured by counting all of the sporangia in the entire field of vision under a microscope at 100 times magnification. To obtain zoospores, the colonies were flooded with 10 mL sterile water and incubated at 4°C for 30 min followed by incubation at room temperature for 30 min. Zoospore production was measured by determining zoospore concentrations using a hemocytometer.

### Nucleic acid isolation and PCR analysis.

Total DNA was isolated using the method detailed in a previous study (46). Total RNA was extracted from the frozen samples with the SV Total RNA isolation kit (Promega, Beijing, China), after which cDNA was synthesized with the PrimeScript RT reagent kit with gDNA Eraser (TaKaRa, Beijing, China) according to the protocols of the manufacturers. The PCR was conducted using 2× Master Mix (Tsingke, Beijing, China) with EasyTaq DNA polymerase. Each 25 μL reaction mixture contained 12.5 μL 2× Master Mix, 0.5 μL forward primer (10 μM), 0.5 μL reverse primer (10 μM), and 500 to 1,000 ng template DNA. Amplification was performed using a PCR thermocycle instrument with a recommended protocol. The resulting PCR products were analyzed with agarose gel electrophoresis and Sanger sequencing.

### Bioinformatic analysis of PcSCPs.

The DNA sequences of the four *PcSCPs* were retrieved from the genome data of *P. capsici* in the JGI database (https://phycocosm.jgi.doe.gov/Phyca11/Phyca11.home.html), with the corresponding protein IDs being 543794, 54680, 563749, and 506722 for *PsSCP1-4*, respectively. The coding sequence of each gene was further refined with the ORF Finder online (http://www.bioinformatics.org/sms2/orf_find.html) and confirmed by reverse transcription-PCR (RT-PCR). For phylogenetic analysis, the protein sequences of SCPs from different eukaryotic lineages were retrieved from the NCBI database, including HMGCR, SCAP, NPC1, PTC, and DISP proteins from Homo sapiens, Bos taurus, Mus musculus, and Gallus gallus. In addition, four homologs of PcSCPs were identified from the genome data of *P. sojae* (18) and verified with the ORF Finder and RT-PCR. The phylogenetic tree was constructed with Mega 6.0 (47). The Simple Modular Architecture Research Tool (SMART, http://smart.embl-heidelberg.de/) was used for functional domain prediction (48). The TMHMM-2.0 (https://services.healthtech.dtu.dk/service.php?TMHMM-2.0) was used for the prediction of transmembrane segments.

### Genetic manipulation and genotype confirmation.

To disrupt the *PcSCPs*, sgRNAs were designed with an online tool (EuPaGDT, http://grna.ctegd.uga.edu/) and cloned into the sgRNA expressing vector, pYF2.3G-ribo-sgRNA, as described in a previous study (49). A novel selection marker, *PcMuORP1*, was then introduced into the sgRNA expressing plasmids (29). For homology-directed repair (HDR)-mediated replacement, the template plasmids were constructed by cloning donor DNA (*NPT II* for *PcSCP1* and *eGFP* for the other *PcSCPs*) along with about 1,000 bp flanking sequences of the knockout portions of *PcSCPs* into pBluescript II KS+. Three plasmids (the sgRNA expressing plasmid, the simplified Cas9 expressing plasmid, and the template plasmid), listed in Table S2, were simultaneously transformed into protoplasts of *P. capsici*, and the potential transformants were screened with oxathiapiprolin as detailed in a previous study (29). The genotypes of the transformants were verified with the primers listed in Table S1. The *P. palmivora* transformants expressing Lifeact-eGFP available at Wageningen University were generated as described previously (50).

### Sterol extraction and detection.

The wild-type strain and one “all-four” *PcSCPs* knockout transformant were cultured on solid minimal medium without sterol for two rounds, before they were transferred into liquid minimal medium modified with 20 μg/mL β-sitosterol, with at least three replicates for each sample. The mycelia were collected after 10 days’ cultivation at 25°C with a rotation frequency of 120 rpm. The collected mycelia were washed three times with double-distilled water and subsequently dehydrated by freeze-drying. Sterol extraction was performed with 0.1 g dried sample using a protocol described in a previous study (15), with 50 μg cholesterol being used as an internal standard for each detection. The derivatization reaction was carried out with N,O-bis(trimethylsilyl)-trifluoroacetamide (BSTFA) and sterol detection was performed via gas chromatography in tandem with a triple quadrupole mass spectrometer (GC-MS, Agilent 7890B-7000C). The sample (1 μL) was injected onto the column (HP-5MS UI, 0.25 μm, 0.25 mm × 15 m). The temperature program was as follows: 80°C for 1 min, followed by a temperature increase of 20°C/min to 270°C (8 min) and another temperature increase of 30°C/min to 300°C (5 min). The data were collected in MRM mode, and the following ion pairs (*m/z*) were used for the detection of different sterols: 458.5 > 368.4 and 368.4 > 353.4 for cholesterol; 396.0 > 172.8 and 357.0 > 120.7 for β-sitosterol.

### Transcriptome analysis.

The wild-type strain and one “all-four” *PcSCPs* knockout transformant that had undergone two rounds of cultivation on minimal medium were transferred into liquid minimal medium modified with 20 μg/mL β-sitosterol and 1% methanol, or to the minimal medium modified with only 1% methanol. The strains were cultured at 25°C in the dark for 4 days, then transferred to the light for another 4 h before the mycelia were collected. RNA sequencing was performed by Shanghai OE Biotech. Co., Ltd. Briefly, total RNA was isolated and treated with DNase, after which the mRNA was enriched using magnetic beads with Oligo (dT). The mRNA was digested into fragments before double-stranded cDNA was synthesized and purified. The cDNA library was constructed via PCR amplification, and the library was sequenced on an Illumina Hiseq 2500 Platform. The RNA-Seq reads were processed with Trimmomatic (51) and clean reads were mapped to the reference genome of *P. capsici* (https://mycocosm.jgi.doe.gov/Phyca11/Phyca11.home.html) using HISAT2 (52). Expression levels of protein-coding genes were calculated using the FPKM method (53). Principal-component analysis (PCA) was used to investigate the transcriptome distance of the samples. GO enrichment analysis of the DEGs was performed taking three aspects into consideration, including biological process, cellular component, and molecular function.

### Microscopy.

For membrane staining of sporangia, the mycelia with sporangia were placed in ice water for 10 min and then treated with 6 μM FM4-64 at room temperature for 5 min. The membrane structures were observed under the laser confocal microscopy (Leica), with an excitation wavelength of 552 nm and an emission wavelength of 562 to 620 nm. For F-actin visualization, rhodamine-phalloidin staining was applied according to the protocol used in a previous study (54) with a few modifications. Sporangia were prefixed in 120 μL of a solution of m-maleimidobenzoyl-N-hydroxysulfosuccinimide ester (MBS ester), which was dissolved in liquid minimal medium to a final concentration of 800 μM. After 5 to 10 min, the samples were fixed in 120 μL of a solution of 4% paraformaldehyde and 0.5% methyglyoxal in 50 mM PIPEs buffer for 30 min. Then, samples were washed twice, 5 min each, in 120 μL of 50 mM PIPEs, incubated in 100 μL of a solution of 0.36 mM rhodamine-phalloidin (Thermo Fisher Scientific) for 30 min in the dark, and again washed twice, 5 min each, in 120 μL of 50 mM PIPEs in the dark. To reduce photobleaching, the samples were enclosed in 20 μL of Vectashield Plus (Vector Laboratories) and observed immediately. Microscopic analyses were performed on the Roper Spinning Disk microscope system (Evry, France) with a Yokogawa spinning disk head, inverted Nikon Eclipse Ti microscope, and Metamorph software.

### Infection assays.

To compare the pathogenicity of the transformants and the wild-type strain, the strains were cultured on V8 medium at 25°C in the dark for 4 days, after which mycelial plugs (5 mm in diameter) were cut from the edges of the colonies and inoculated on the abaxial side of detached pepper leaves, with five replicates for each strain. After 3 days of incubation at 25°C in the dark with 80% humidity, lesion sizes were measured.

### Statistical analysis.

The data collected were subjected to analysis of variance using DPS software ver. 7.05. The number of replicates used for each experiment is indicated in the corresponding figure legends. Differences between means of different treatments were determined using Duncan’s multiple range test at *P = *0.01.

### Data accessibility.

The RNA-seq data have been deposited in the NCBI Sequence Read Archive database under the accession number PRJNA900211.
