# Supplementary material for: Sterol-Sensing Domain (SSD)-Containing Proteins in Sterol Auxotrophic Phytophthora capsici Mediate Sterol Signaling and Play a Role in Asexual Reproduction and Pathogenicity
Source: Microbiol Spectr. 2023 Jan 11;11(1):e03797-22. doi: 10.1128/spectrum.03797-22 (PMC9927452; doi:10.1128/spectrum.03797-22)
Supplement: Supplemental file 1 — Supplemental material. Download spectrum.03797-22-s0001.pdf, PDF file, 1.1 MB [file spectrum.03797-22-s0001.pdf]

Table S1 Primers used in this study

Primer	Sequence 5′-3′	Purpose
SCP1-F	ATGGACTGCCCCTTGCTG	Amplification of the whole gene of *PcSCP1*
SCP1-R	TCAGTACTTGCCGGTAGGAGG	
SCP2-F	ATGCTCTGCTGTACAGAGTC	Amplification of the whole gene of *PcSCP2*
SCP2-R	CTACTCGACACGAGTGACAGGG	
SCP3-F	ATGGCGCTGTCTGCGCTCGT	Amplification of the whole gene of *PcSCP3*
SCP3-R	CTAGCGCACTGCTTTGAAGA	
SCP4-F	ATGCGCGTCTCGCTGCTGCT	Amplification of the whole gene of *PcSCP4*
SCP4-R	TTAGTCGACGCGCGTCACAG	
S1-up-F	ATCGATAAGCTTGATCGTGGATGCCAATTACGTT	Amplification of the upstream sequence of the knockout part of *PcSCP1* for template plasmid construction
S1-up-R	ATCTTGTTCAATCATCTGGAACTTCTCCTGGTTC	
S1-NPT-F	ATGATTGAACAAGATGGATTGCACGCAGGTTCTCC	Amplification of the donor DNA *NPT II* for template plasmid construction for *PcSCP1* knocking out
S1-NPT-R	GAGCCACTGCGCTGCTCAGAAGAACTCGTCAAGAA	
S1-down-F	GCAGCGCAGTGGCTCGCT	Amplification of the downstream sequence of the knockout part of *PcSCP1* for template plasmid construction
S1-down-R	CTGCAGGAATTCGATTTACCGGAACAGGTTGACT	
S2-up-F	ATCGATAAGCTTGATACACAATGATTGCCCAGCC	Amplification of the upstream sequence of the knockout part of *PcSCP2* for template plasmid construction
S2-up-R	CTCGCCCTTGCCCATATCGTCACCCATAGCGATA	
S2-GFP-F	ATGGGCAAGGGCGAGGAACTGTTCACTGGCGTGG	Amplification of the donor DNA *eGFP* for template plasmid construction for *PcSCP2* knocking out
S2-GFP-R	AACAGACTTGGGTTATCAAGGCGCGCCTGCGGC	
S2-down-F	TAACCCAAGTCTGTTTCAGTTGCTTTTCATTAGC	Amplification of the downstream sequence of the knockout part of *PcSCP2* for template plasmid construction
S2-down-R	CTGCAGGAATTCGATCAACTCGGCCGAGAACTT	
S3-up-F	ATCGATAAGCTTGATGGGATGACTGCTCCTGGG	Amplification of the upstream sequence of the knockout part of *PcSCP3* for template plasmid construction
S3-up-R	CTCGCCCTTGCCCATTAGCACGGGGAACTCGCT	
S3-GFP-F	ATGGGCAAGGGCGAGGAACTGTTCACTGGCGTGG	Amplification of the donor DNA *eGFP* for template

S3-GFP-R	TTTTCATTGTTTCGTTCAAGGCGC GCCTGCGGC	plasmid construction for *PcSCP3* knocking out
S3-down-F	ACGAAACAATGAAAAAATGAACG TGGTAATAATG	Amplification of the downstream sequence of the knockout part of *PcSCP3* for template plasmid construction
S3-down-R	CTGCAGGAATTCGATTGGACGGA ACTGGTAGGG	
S4-up-F	ATCGATAAGCTTGATTGCTCTCCA CCGGTTGTT	Amplification of the upstream sequence of the knockout part of *PcSCP4* for template plasmid construction
S4-up-R	CTCGCCCTTGCCCATCCGGTTGTT CATTGGCAG	
S4-GFP-F	ATGGGCAAGGGCGAGGAACTGTT CACTGGCGTGG	Amplification of the donor DNA *eGFP* for template plasmid construction for *PcSCP4* knocking out
S4-GFP-R	GCTTGGGCTGCCAACTCAAGGCGC GCCTGCGGC	
S4-down-F	GTTGGCAGCCCAAGCCGCTTGCAT TCATGTCTCC	Amplification of the downstream sequence of the knockout part of *PcSCP4* for template plasmid construction
S4-down-R	CTGCAGGAATTCGATTCGCGAGCC ACGGATTCG	
KS1-F	CAGGTGGTTCTTCTTTATGAGG	Confirmation of the occurance of the replacement of *PcSCP1*
KS1-R	AATAGCAGCCAGTCCCTTC	
KS2-F	ATTCAAGCTGACAGCGTAAG	Confirmation of the occurance of the replacement of *PcSCP2*
KS2-R	GTAGTTCCCGTCATCCTTGA	
KS3-F	TCGTCTCTGGCTTGGGCTAC	Confirmation of the occurance of the replacement of *PcSCP3*
KS3-R	GTAGTTCCCGTCATCCTTGA	
KS4-F	CGTTGCACTTCTGATCTCGG	Confirmation of the occurance of the replacement of *PcSCP4*
KS4-R	GTAGTTCCCGTCATCCTTGA	
S1-F	ATGGACTGCCCCTTGCTGTAC	Checking the homozygosity of *PcSCP1* knockout transformants
S1-R	TGGCATAGCTGAATACGCTG	
S2-F	AGTACTTCCAGAACAACATG	Checking the homozygosity of *PcSCP2* knockout transformants
S2-R	ATACGATGATGGTTGTGATC	
S3-F	TCGTGCTGCTACGGCGTTTC	Checking the homozygosity of *PcSCP3* knockout transformants
S3-R	CTAGCGCACTGCTTTGAAGA	
S4-F	ATGACGTCCTGTGTGGGCGT	Checking the homozygosity of *PcSCP4* knockout transformants
S4-R	TCATCGTCCGACTTGTACAC	
sgS1-F	CTAGCACCTGCCTGATGAGTCCGT GAGGACGAAACGAGTAAGCTCGT C**GCAGGTTAACTTCACGACGA**	sgRNA expressing plasimid construction for *PcSCP1*

sgS1-R	AAACTCGTCGTGAAGTTAACCTGCGACGAGCTTACTCGTTTCGTCCTCACGGACTCATCAGGCAGGTG	
sgS2-F	CTAGCGCCAACCTGATGAGTCCGTGAGGACGAAACGAGTAAGCTCGTC**GTTGGCCTCCTGCTGATTGT**	sgRNA expressing plasimid construction for *PcSCP2*
sgS2-R	AAACACAATCAGCAGGAGGCCAACGACGAGCTTACTCGTTTCGTCCTCACGGACTCATCAGGTTGGCG	
sgS3-F	CTAGCATCCCACTGATGAGTCCGTGAGGACGAAACGAGTAAGCTCGTC**TGGGATCATTCAGCGTTTCG**	sgRNA expressing plasimid construction for *PcSCP3*
sgS3-R	AAACCGAAACGCTGAATGATCCCAGACGAGCTTACTCGTTTCGTCCTCACGGACTCATCAGTGGGATG	
sgS4-F	CTAGCTCTCGCCTGATGAGTCCGTGAGGACGAAACGAGTAAGCTCGTC**GCGAGATGTAAATGATCATC**	sgRNA expressing plasimid construction for *PcSCP4*
sgS4-R	AAACGATGATCATTTACATCTCGCGACGAGCTTACTCGTTTCGTCCTCACGGACTCATCAGGCGAGAG	
RNASeqQ1-F	TCAAGGCGAATGTTGTGCAG	qPCR of the gene 10089 for transcriptome validation
RNASeqQ1-R	ACCGTTTGGCTTGGCATTTG	
RNASeqQ2-F	TGCACTTGCGCTGAAATTCG	qPCR of the gene 102025 for transcriptome validation
RNASeqQ2-R	TTTCGCGCTTTTGCTCCATG	
RNASeqQ3-F	ACGCTTTCAACGACAGCAAC	qPCR of the gene 102833 for transcriptome validation
RNASeqQ3-R	AAAGCTTGCCAGTCGAGATC	
RNASeqQ4-F	ACGCTTTTGTCTCCGCAAAG	qPCR of the gene 104320 for transcriptome validation
RNASeqQ4-R	TTGCAAACACGCGTTCGATG	
RNASeqQ5-F	TGCGTTGTTGCTGCTATTGC	qPCR of the gene 106493 for transcriptome validation
RNASeqQ5-R	TTGGAAACGAGGTGAATGCG	
RNASeqQ6-F	TTTCCGTCGCGAAACAACTG	qPCR of the gene 107237 for transcriptome validation
RNASeqQ6-R	TGCATTGGTTGGACAACGAC	
RNASeqQ7-F	ACTACCAGCCCAACTAAAGCC	qPCR of the gene 107323 for transcriptome validation
RNASeqQ7-R	ATTTCTGTGCGTGGGTTTGC	
RNASeqQ8-F	AAGCCAATGGAAACGTGTGG	qPCR of the gene 109154 for transcriptome validation
RNASeqQ8-R	TGCAATTCATCCGTGGTAGC	
RNASeqQ9-F	AATGCGTGCACTCAAAGAGC	qPCR of the gene 115142 for transcriptome validation
RNASeqQ9-R	TCTTGTCGATGCAACCAACG	

RNASeqQ10-F	TGCCTTCATGAACCCGAATG	qPCR of the gene 118061 for transcriptome validation
RNASeqQ10-R	AAAGCAAGGCCTTTGAGCAC	
RNASeqQ11-F	AAAATCGACGGCACTGTGAC	qPCR of the gene 133008 for transcriptome validation
RNASeqQ11-R	ACTTGGCTGACTGGACTTTG	
RNASeqQ12-F	AGCACAACCGGTTTCTTGAG	qPCR of the gene 15283 for transcriptome validation
RNASeqQ12-R	AGCATGCGTCATTGTTTGCC	
RNASeqQ13-F	TGTGAACATGACTGCAGTGC	qPCR of the gene 17044 for transcriptome validation
RNASeqQ13-R	TAAACGCACGATGGCTCTTG	
RNASeqQ14-F	TTCCACTGCTCAAGGACAAGG	qPCR of the gene 4291 for transcriptome validation
RNASeqQ14-R	GTCTTCAATGCACGCATTCG	
RNASeqQ15-F	TCAAATTGCGCCACAACTCG	qPCR of the gene 505255 for transcriptome validation
RNASeqQ15-R	TTCACCTCGAGCAGCAATTG	
RNASeqQ16-F	ACGCGTACAAACTCATTGGC	qPCR of the gene 506723 for transcriptome validation
RNASeqQ16-R	TAGCTGTCAACGCAATCACC	
RNASeqQ17-F	TCACGTGTGATAGCAAGACG	qPCR of the gene 529263 for transcriptome validation
RNASeqQ17-R	TCGTTTCGCTTTGGATGTGC	
RNASeqQ18-F	ATGTCCTGAACGTGGTTTGG	qPCR of the gene 538368 for transcriptome validation
RNASeqQ18-R	ACAGCTTTCTGCCTGCCATAG	
RNASeqQ19-F	TCGGAAGCTCCAAATTGCTC	qPCR of the gene 100849 for transcriptome validation
RNASeqQ19-R	TTTCGCTCGCATTTCCTTCG	
RNASeqQ20-F	TGCTCACAAACTCCAACACG	qPCR of the gene 113574 for transcriptome validation
RNASeqQ20-R	ATGTGTCGGTTTCGCAGATG	
RNASeqQ21-F	AAACGCGTGACTTGCCAAAG	qPCR of the gene 121635 for transcriptome validation
RNASeqQ21-R	AATGCCTGCACTGCTGATTG	
RNASeqQ22-F	ACGTGGCTTGTAAACAGTGC	qPCR of the gene 509844 for transcriptome validation
RNASeqQ22-R	AACTTGTGGTGCCAGTTGTG	
RNASeqQ23-F	TCGGTCCGTTTTCAAGCAAC	qPCR of the gene 532602 for transcriptome validation
RNASeqQ23-R	AAACCGGTGTAGTTCTTGCC	
WS21-F	GGAAAGAACAAACGCCTGAC	Amplification of the reference gene *WS21* for qPCR
WS21-R	GTTGCGCTCCGAGAAGATA	

Bold nucleotides indicate the sgRNA sequence, and underlined nucleotides indicate the overlapping sequences required to join DNA fragments during In-Fusion® HD cloning.

Table S2 Plasmids used for knocking out *PcSCPs*

Target gene	Gene length	Number of introns	Length of deletion	Plasmids used
PcSCP1	4146 bp	0	2327 bp	pYF2.3G-PcMuORP1-S1<br>pYF-Cas9-EI<br>pB-S1-NPT
PcSCP2	3981 bp	0	3981 bp	pYF2.3G-PcMuORP1-S2<br>pYF-Cas9-EI<br>pB-S2-GFP
PcSCP3	3093 bp	4	3093 bp	pYF2.3G-PcMuORP1-S3<br>pYF-Cas9-EI<br>pB-S3-GFP
PcSCP4	4454 bp	1	3488 bp	pYF2.3G-PcMuORP1-S4<br>pYF-Cas9-EI<br>pB-S4-GFP

Table S3 qPCR confirmation of the relative expression of the DEGs identified in the transcriptome analyses

Comparison group	Gene ID [a]	Change type in transcriptome	Change fold in transcriptome [b]	Change fold by qPCR
1	10089	up	22.67	12.32
1	102025	up	15.62	11.96
1	102833	up	55.72	3.26
1	104320	up	52.89	10.65
1	106493	up	11.97	4.45
1	107237	up	#	1.84
1	107323	up	16.21	8.08
1	109154	up	3.03	1.23
1	115142	up	#	20.44
1	118061	up	18.42	20.92
1	133008	up	#	11.66
1	4291	up	26.13	9.56
1	529263	up	#	0.91
1	538368	up	#	9.04
1	15283	down	0.20	0.22
1	17044	down	0.12	0.18
1	505255	down	0.32	0.52
1	506723	down	0.08	0.27
2	100849	up	20.85	1.56
2	113574	up	2.70	2.71
2	509844	up	8.10	4.86
2	532602	up	4.39	14.55
2	121635	down	0.45	0.73

[a] The Gene ID corresponds with the protein ID in the JGI database; [b] "#" indicates infinite fold of change due to the lack of expression in the transcriptome of sample that was not treated with sterol. Comparison group 1: Sample_WT_DO-vs-Sample_WT_CK; Comparison group 2: Sample_Nu14_DO-vs-Sample_Nu14_CK.

Table S4 DEGs related to pathogenesis based on GO classifictions

Comparison	GO term	Gene ID	Fold of change	Regulation
PcSCPs knockout transformant KS2/4/3/1-14-vs-wild type strain BYA5 without sterol	extracellular region|defense response|pathogenesis	PHYCA_62714	0.1858	down
		PHYCA_571374	0.1973	down
		PHYCA_508761	0.0357	down
		PHYCA_508763	0.1122	down
		PHYCA_508766	0.0282	down
		PHYCA_508770	0.0186	down
		PHYCA_506685	13.4510	up
		PHYCA_566444	4.8468	up
		PHYCA_109170	0.0312	down
		PHYCA_116840	0.1277	down
		PHYCA_567552	0.0225	down
		PHYCA_570597	0.0527	down
		PHYCA_16638	3.5232	up
		PHYCA_503459	0.3559	down
PcSCPs knockout transformant KS2/4/3/1-14-vs-wild type strain BYA5 with sterol	extracellular region|defense response|pathogenesis	PHYCA_571374	0.0627	down
		PHYCA_549042	0.1577	down
		PHYCA_11949	0.0819	down
		PHYCA_508770	0.3625	down
		PHYCA_109170	0.1211	down
		PHYCA_116840	0.1433	down
		PHYCA_570597	0.1827	down
		PHYCA_109069	0.0712	down
		PHYCA_511538	0.0244	down

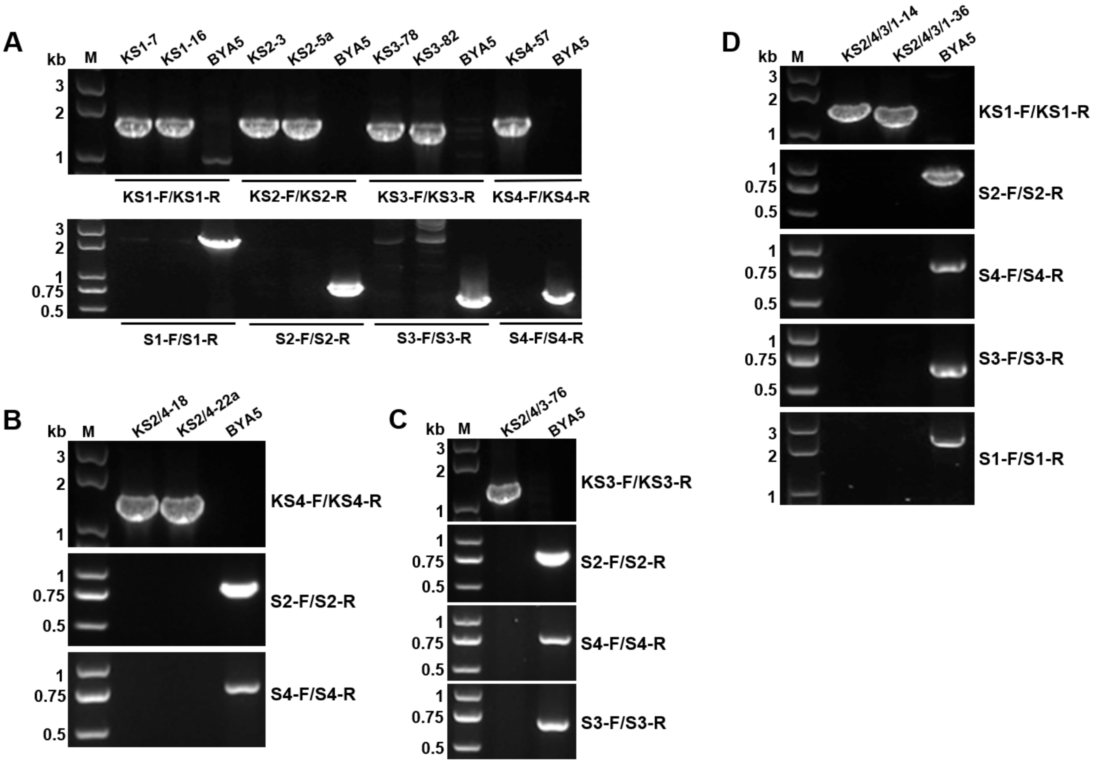

FIG S1 Genotype validation of the knockout transformants of *P. capsici*. (A) Confirmation of the single *PcSCP* knockout transformants. BYA5 is the wild-type strain; KS1-7 and KS1-16 are *PcSCP1* knockout transformants; KS2-3 and KS2-5a are *PcSCP2* knockout transformants; KS3-78 and KS3-82 are *PcSCP3* knockout transformants; KS4-57 is a *PcSCP4* knockout transformant. (B) Confirmation of the double knockout transformants. KS2/4-18 and KS2/4-22a are the *PcSCP2/4* knockout transformants. (C) Confirmation of the triple knockout transformant. KS2/4/3-76 is a *PcSCP2/4/3* knockout transformant. (D) Confirmation of the "all four" knockout transformants. KS2/4/3/1-14 and KS2/4/3/1-36 are the *PcSCP2/4/3/1* knockout transformants. In (A), (B), (C) and (D), on the top of each panel is shown the amplification results with replacement specific primers (KS1-F/KS1-R for *PcSCP1* replacement, KS2-F/KS2-R for *PcSCP2* replacement, KS3-F/KS3-R for *PcSCP3* replacement, and KS4-F/KS4-R for *PcSCP4* replacement). Target gene specific primers (S1-F/S1-R for *PcSCP1*, S2-F/S2-R for *PcSCP2*, S3-F/S3-R for *PcSCP3*, and S4-F/S4-R for *PcSCP4*) were used for the detection of the presence of the target genes. All the PCR products have the predicted lengths.

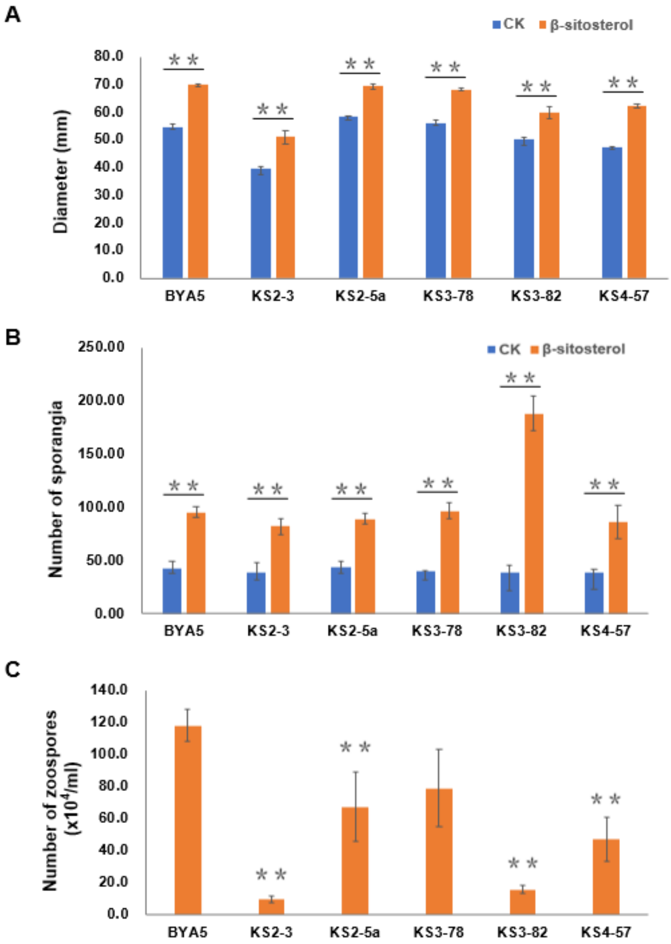

FIG S2 Effects of β-sitosterol on wild-type strain and single *PcSCP* knockout transformants of *P. capsici*. The mycelial growth (A) and sporangium production (B) of the wild-type strain and single *PcSCP* knockout transformants cultured on the minimal medium without sterol (CK) or modified 20 μg/ml β-sitosterol. Means with SD of three replicates are shown, and double asterisks denote a significant difference from each other (**, $P < 0.01$). (C) The zoospore production of the wild-type strain and single *PcSCP* knockout transformants cultured on the minimal medium modified 20 μg/ml β-sitosterol. Means with SD of three replicates are shown, and double asterisks denote a significant difference from the wild-type strain (**, $P < 0.01$). BYA5 is the wild-type strain; KS2-3 and KS2-5a are *PcSCP2* knockout transformants; KS3-78 and KS3-82 are *PcSCP3* knockout transformants; KS4-57 is a *PcSCP4* knockout transformant. CK means the strains were cultured without sterol treatment and zoospore production was measured under β-sitosterol treatment.

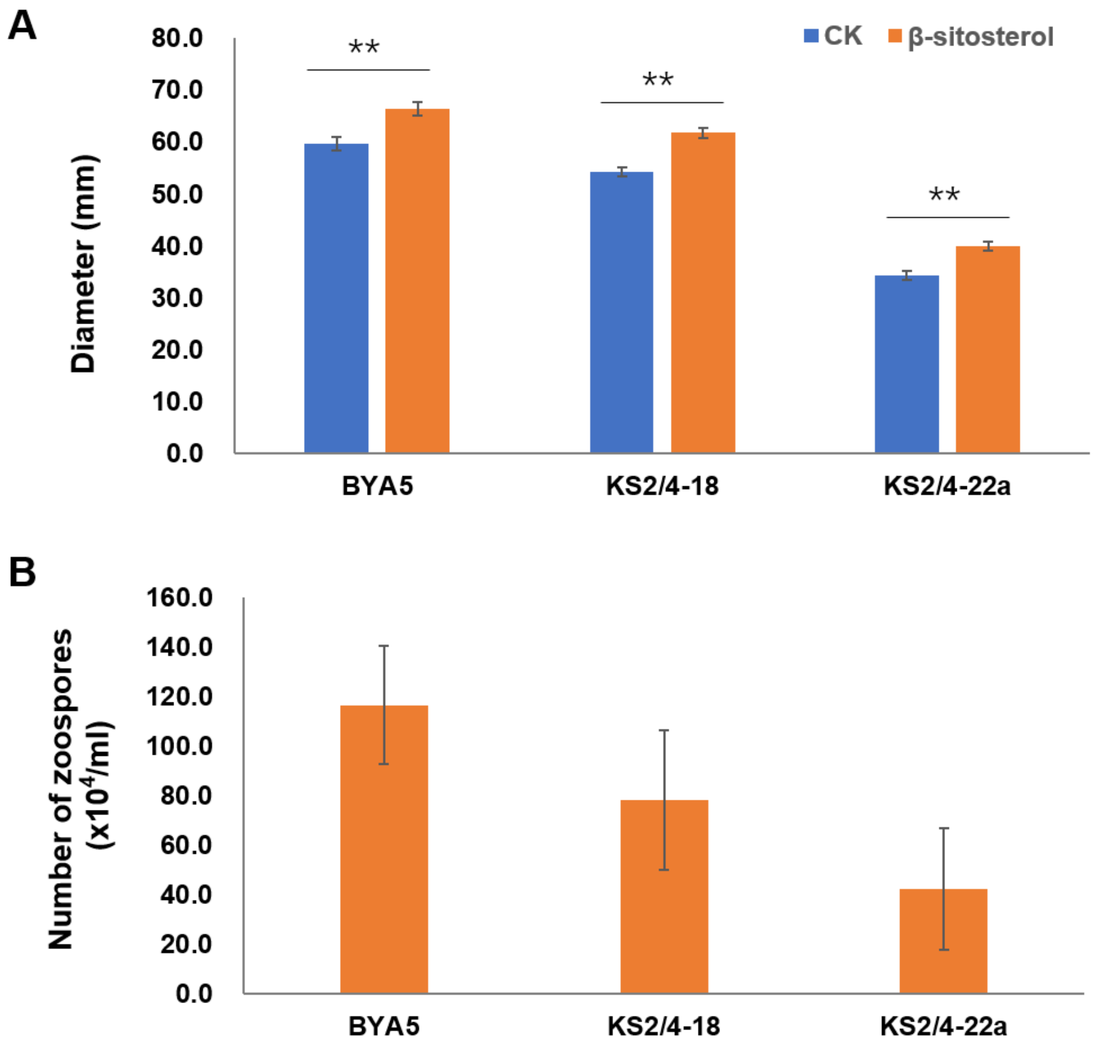

FIG S3 Effects of β-sitosterol on wild-type strain and double *PcSCPs* knockout transformants of *P. capsici*. (A) The mycelial growth the wild-type strain and double *PcSCPs* knockout transformants cultured on the minimal medium without sterol (CK) or modified 20 μg/ml β-sitosterol. Means with SD of three biological replicates of one experiment are shown, and double asterisks denote a significant difference from each other (**, $P < 0.01$). (B) The zoospore production of the wild-type strain and *PcSCPs* knockout transformants cultured on the minimal medium modified 20 μg/ml β-sitosterol. Means with SD of three replicates are shown. BYA5 is the wild-type strain; KS2/4-18 and KS2/4-22a are *PcSCP2/4* knockout transformants. CK means the strains were cultured without sterol treatment and zoospore production was measured under β-sitosterol treatment.

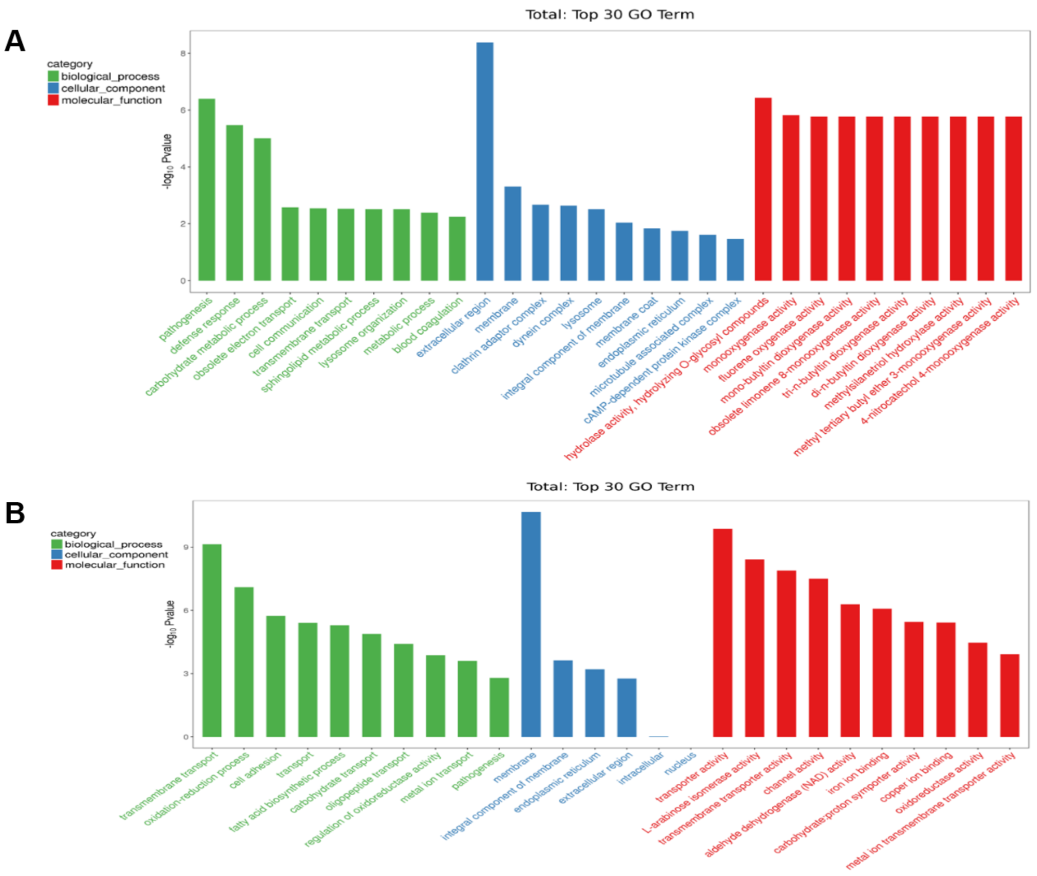

FIG S4 Gene ontology (GO) annotation of genes that showed changes in expression in the transcriptome. (A) Comparison group 1 (Sample_WT_DO-vs-Sample_WT_CK) displaying the wild-type strain BYA5 with sterol treatment compared to BYA5 without sterol treatment. (B) Comparison group 2 (Sample_Nu14_DO-vs-Sample_Nu14_CK) displaying the *ΔPcSCP2/4/3/1* transformant KS2/4/3/1-14 with sterol treatment compared to KS2/4/3/1-14 without sterol treatment. The number of genes with different GO terms is shown in the three GO categories: biological process, cellular component and molecular function.

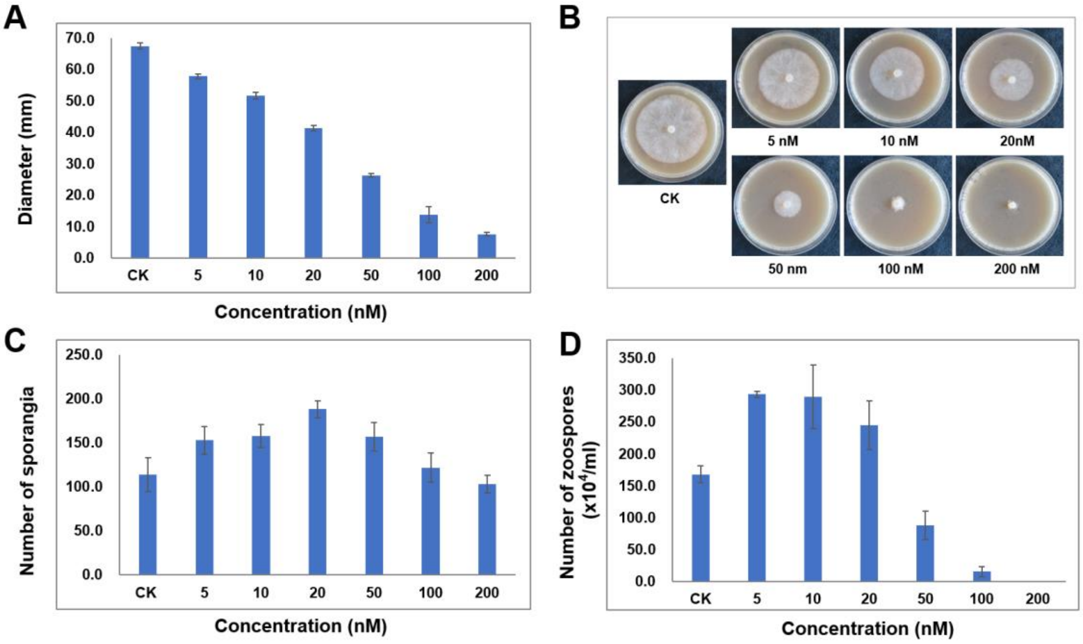

FIG S5 Influence of latrunculin B on the growth and development of *P. capsici*. Influence of latrunculin B on mycelial growth (A, B), sporangium production (C) and oospore production (D). The *P. capsici* wild-type strain BYA5 was cultured on V8 medium modified with different concentrations of latrunculin B, with normal V8 medium without latrunculin B as a control. Means with SD of three replicates are shown.

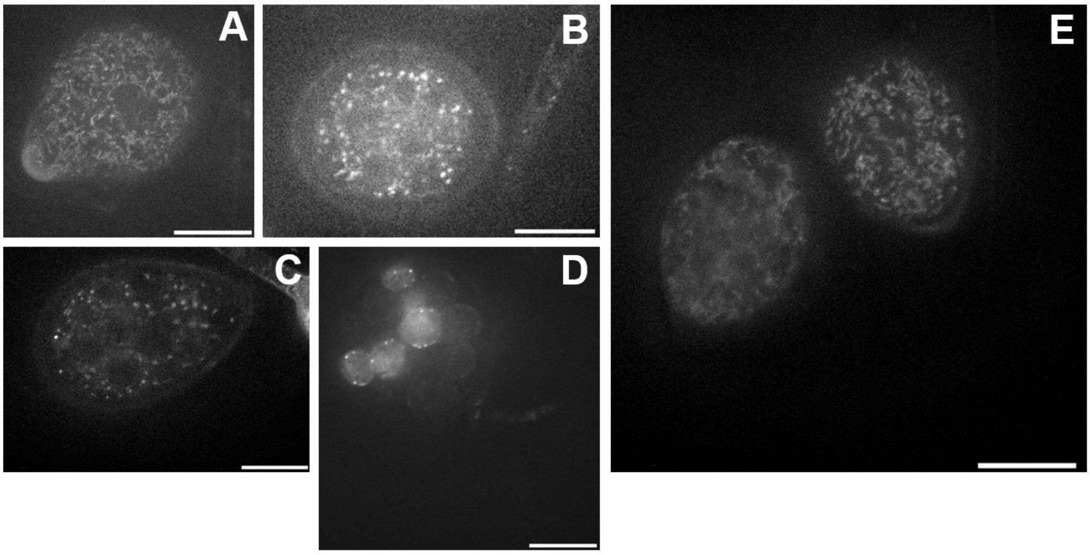

FIG S6 F-actin organization in sporangia of *P. palmivora* in the absence or presence of sterol. labelled with Lifeact-eGFP under sterol treatment or not. Fluorescence live cell imaging was conducted with a *P. palmivora* transformant expressing Lifeact-eGFP. F-actin configuration before (A), during (B, C), and after zoospore cleavage (D) in sporangia cultured in the presence of 20 μg/ml β-sitosterol. (E) F-actin configuration in sporangia cultured without sterol. In the absence of sterols there is no zoosporogenesis and no zoospore cleavage. Bar = 20 μm.

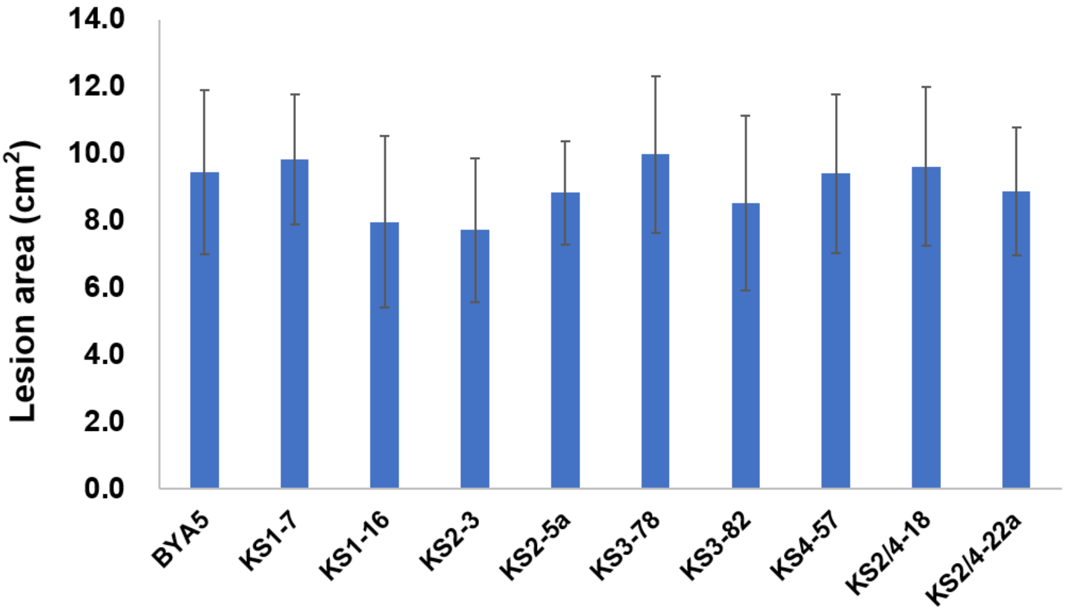

FIG S7 Pathogenicity evaluation of the wild-type strain and *PcSCPs* knockout transformants with pepper leaves. Detached pepper leaves were inoculated with mycelial plugs and the lesions areas were determined at 3 dpi. Means with SD of five replicates of one experiment are shown. BYA5 is the wild-type strain; KS1-7 and KS1-16 are *PcSCP1* knockout transformants; KS2-3 and KS2-5a are *PcSCP2* knockout transformants; KS3-78 and KS3-82 are *PcSCP3* knockout transformants; KS4-57 is a *PcSCP4* knockout transformant; KS2/4-18 and KS2/4-22a are the *PcSCP2/4* knockout transformants.
